# In vitro antibacterial activity of rifampicin in combination with imipenem, meropenem and doripenem against multidrug-resistant clinical isolates of *Pseudomonas aeruginosa*

**DOI:** 10.1186/s12879-016-1785-7

**Published:** 2016-08-24

**Authors:** Yi-Fan Hu, Chang-Pan Liu, Nai-Yu Wang, Shou-Chuan Shih

**Affiliations:** 1 Division of Infectious Diseases, Department of Internal Medicine, MacKay Memorial Hospital, Taipei, Taiwan; 2 Department of Medical Research, MacKay Memorial Hospital, No. 92, Sec. 2, Zhongshan N. Road, Taipei, Taiwan; 3 Department of Medicine, MacKay Medical College, New Taipei City, Taiwan; 4 MacKay College of Medicine, Nursing and Management, Taipei, Taiwan; 5 Infection Control Committee, MacKay Memorial Hospital, Taipei, Taiwan; 6 Division of Gastroenterology, Department of Internal Medicine, MacKay Memorial Hospital, Taipei, Taiwan

**Keywords:** Frameshift mutation, Imipenem, Porin mutation, *Pseudomonas aeruginos*a, Rifampicin

## Abstract

**Background:**

Multidrug-resistant *Pseudomonas aeruginosa* has emerged as one of the most important healthcare-associated pathogens. Colistin is regarded as the last-resort antibiotic for multidrug-resistant Gram-negative bacteria, but is associated with high rates of acute kidney injury. The aim of this in vitro study is to search for an alternative treatment to colistin for multidrug-resistant *P. aeruginosa* infections.

**Methods:**

Multidrug and carbapenem-resistant *P. aeruginosa* isolates were collected between January 2009 and December 2012 at MacKay Memorial Hospital. Minimal inhibitory concentrations (MICs) were determined for various antibiotic combinations. Carbapenemase-producing genes including *bla*_VIM,_ other β-lactamase genes and porin mutations were screened by PCR and sequencing. The efficacy of carbapenems (imipenem, meropenem, doripenem) with or without rifampicin was correlated with the type of porin mutation (frameshift mutation, premature stop codon mutation) in multidrug-resistant *P. aeruginosa* isolates without carbapenemase-producing genes.

**Results:**

Of the 71 multidrug-resistant clinical *P. aeruginosa* isolates, only six harboured the *bla*_VIM_ gene. Imipenem, meropenem and doripenem were significantly more effective (reduced fold-change of MICs) when combined with rifampicin in *bla*_VIM_-negative isolates, especially in isolates with porin frameshift mutation.

**Conclusions:**

Imipenem + rifampicin combination has a low MIC against multidrug-resistant *P. aeruginosa,* especially in isolates with porin frameshift mutation. The imipenem + rifampicin combination may provide an alternative treatment to colistin for multidrug -resistant *P. aeruginosa* infections, especially for patients with renal insufficiency.

## Background

*Pseudomonas aeruginosa* is one of the leading pathogens causing healthcare-associated infections. Besides being innately resistant to a myriad of antibiotics used to treat Gram-negative infections, a number of *P. aeruginosa* isolates has been acquiring multidrug resistance (MDR) at an alarming rate, raising much clinical concern. Carbapenems are an important class of antimicrobial agents used to treat *P. aeruginosa* infections [1]; as such, the acquisition of resistance against carbapenems in many *P. aeruginosa* isolates is especially worrisome.

Development of multidrug resistance in *P aeruginosa* is common, especially when antibiotics exert strong selective pressure on bacterial populations [2, 3]. The resistant mechanisms of multidrug-resistant *P. aeruginosa* include acquisition of carbapenemase gene, inactivation of *oprD* causing outer-membrane impermeability, and expression of broadly specific multidrug efflux pump systems [2, 3]. Resistance to carbapenem is commonly observed among *P. aeruginosa* isolates and is frequently associated with decreased expression or loss of function of *oprD*, which leads to outer-membrane impermeability [2, 4]. Reduced permeability due to loss of *oprD* leads to a four- to 16-fold increase in the minimum inhibitory concentrations (MICs) for carbapenems in *P. aeruginosa* [3, 4].

Polymyxin antibiotics have been used clinically since the 1960′s and exert activity against many MDR Gram-negative bacteria in vitro, including *P. aeruginosa* and *Acinetobacter baumannii*. Currently, two polymyxin antibiotics are commercially available for clinical use - colistin and polymyxin B - which differ in structure by only one amino acid [5]. Carbapenems are usually prescribed for severe *P. aeruginosa* infections; however, colistin is the only antibacterial agent that currently exerts activity against *P. aeruginosa* strains that are highly resistant to carbapenems [6]. However, nephrotoxicity is a major dose-limiting adverse effect of both polymyxin B and colistin, with rates of acute kidney injury ranging from 30 to 60 % as reported in recent studies [7–9]. The potential nephrotoxicity of colistin is a clinical concern, especially in patients with renal insufficiency.

Although imipenem inhibits most bacterial growth at very low concentrations, some *P. aeruginosa* strains are resistant or become resistant after exposure [10]. Combined antibiotic therapy for invasive *P. aeruginosa* is used in many health care facilities [10, 11]. In vitro studies suggest that rifampicin-based regimens exert synergistic activity when used as part of a combination therapy regimen against carbapenemase-producing *Escherichia coli* and *Klebsiella pneumoniae* [12]. Rifampicin acts to inhibit bacterial DNA-dependent RNA polymerase, which suppresses initial chain formation during RNA synthesis. Alterations to the beta subunit of bacterial DNA-dependent RNA polymerase result in resistance to rifampicin.

The aim of this study was to search for an alternative, combined treatment for multidrug-resistant *P. aeruginosa* infections, in order to avoid the use of colistin and therefore prevent acute kidney injury, especially in patients with renal insufficiency. We assessed the effects of various combinations of antimicrobial agents on multidrug-resistant clinical *P. aeruginosa* isolates. This in vitro data may be useful for supporting therapeutic decisions for patients with severe infections caused by multidrug-resistant *P. aeruginosa.*

## Methods

### Collection of bacterial isolates

With the approval of the Institutional Review Board (protocol number 13MMHIS218), clinical isolates of multidrug and carbapenem-resistant *P. aeruginosa* as identified by the Vitek 2 system ((bioMérieux Vitek Systems Inc., Hazelwood, MO, USA) were collected at MacKay Memorial Hospital, a 2200-bed tertiary teaching hospital in Taiwan, between January 2009 and December 2012. The isolates were confirmed as *P. aeruginosa* using the Vitek 2 system again in a microbiology laboratory. Multidrug resistance is defined as resistance to three or more classes of antibiotics. Carbapenem resistance is defined as minimal inhibitory concentration(MIC) of imipenem ≥ 8 mg/L in accordance with Clinical and Laboratory Standards Institute (CLSI) guidelines [13]. Isolates were stored in trypticase soy broth (BD, MD, USA) containing 20 % glycerol (*v/v*) under −70 °C until further analysis.

### Estimated glomerular filtration rate (eGFR) and creatinine (Cr) level

Patients were classified according to estimated glomerular filtration rate (eGFR) and creatinine (Cr) levels. An estimated glomerular filtration rate ≥ 60 mL/min was classified as eGFR level 1 group; those between 30 to 60 mL/min (30 mL/min ≤ eGFR < 60 mL/min) was classified as level 2, and those <30 mL/min was classified as level 3. Cr level 1 group was defined as serum creatinine level less than 1.5 mg/dL; Cr level 2 group was between 1.5 and 3 (1.5 mg/dL ≤ Cr < 3 mg/dL), and the Cr level 3 group was defined as a serum creatinine level greater than or equal to 3 (Cr ≥3 mg/dL). Renal insufficiency was defined as an eGFR of less than 60 mL/min.

### Antimicrobial susceptibility testing

The antimicrobial susceptibility test of all 71 clinical isolates was determined both by an automated method performed by Vitek2 system and by manual agar dilution method [14]. In the agar dilution method, the effect of individual antibiotics was measured in different concentrations, including 0.03–128 mg/L of ceftazidime, 0.03–128 mg/L of imipenem, 0.03–128 mg/L of meropenem and 0.03–128 mg/L of doripenem. The effect of various combinations of antibiotics was measured by the addition of 4 mg/L tazobactam, 8 mg/L phosphomycin, 8 mg/L sulbactam, 10 mg/L rifampicin, or 20 mg/L rifampicin to various concentrations of ceftazidime, imipenem, meropenem, and doripenem. The MICs were interpreted according to CLSI guidelines [13].

### Phenotypic detection of production of carbapenemase

The production of carbapenemase were screened by the Carba NP test [15]. The Carba NP test is faster and more specific than the modified Hodge test [13], and is therefore more convenient and rapid in the clinical setting.

Briefly, 30 μL of the supernatants of the enzymatic bacterial suspension was mixed with 100 μL aliquots of a 1 mL solution containing 3 mg imipenem monohydrate (USP; Twinbrook Parkway, Rockville, MD, USA), phenol red solution (Merck Millipore, Billerica, MA, USA) and 0.1 mmol/L ZnSO_4_ (Merck Millipore) at pH 7.8. The phenol red solution was prepared by mixing 2 mL of a phenol red solution 0.5 % (wt/vol) with 16.6 mL of distilled water. The mixtures were incubated at 37 °C for a maximum of 2 h. Red or red-orange of Carba NP test was interpreted as negative while yellow or light orange was interpreted as a positive result.

### Phenotypic detection of hyperexpression of efflux pumps and cephalosporinase activity

Imipenem, meropenem and doripenem MIC values were determined in the presence of the efflux pump inhibitor phenyl-arginine-β-naphthylamide (PAβN; at 100 mg/L) and the cephalosporinase (AmpC) inhibitor cloxacillin (at 250 mg/L) [1].

### Polymerase chain reaction and sequencing

The *P. aeruginosa* isolates were screened for carbapenemase-producing genes *bla*_IMP_, *bla*_VIM_, *bla*_NDM_, *bla*_SPM_, *bla*_AIM_, *bla*_DIM_, *bla*_GIM_, *bla*_SIM_, *bla*_KPC_, *bla*_BIC_, *bla*_OXA-48_, Class D genes (*bla*_OXA-group I_, *bla*_OXA-group II_ and *bla*_OXA-group III_) [16] and *oprD* gene mutations [17] using polymerase chain reaction (PCR) and sequencing. Briefly, the bacterial isolates were boiled in sterile water for 10 min, and the supernatants were used for PCR; each 25 μL 2× Hot Master Mix (JMR, Sevenoaks Kent, UK) consisted of 1× S-T Gold buffer, 1.5 mM MgCl_2_, 0.2 mM dNTPs and 20 pmol of each primer. The PCR amplicons were purified using ExoSAP-IT reagent (USB, Cleveland, OH, USA) and both strands were sequenced using the standard dideoxynucleotide method in an ABI Prism 377 DNA sequencer (Applied Biosystems, Foster City, CA, USA). Sequence similarity searches were performed with the basic local alignment search tool (BLAST; http://blast.ncbi.nlm.nih.gov/Blast.cgi).

### Pulsed-field gel electrophoresis

The isolates of 71 multidrug-resistant *P. aeruginosa* were typed by pulsed-field gel electrophoresis (PFGE) following digestion of intact genomic DNA with *Spe*I (Biolabs, Beverly, MA, USA). The DNA fragments were separated on 1 % (*w/v*) SeaKem GTG agarose gels in 0.5 % Tris-borate-ethylene diamine tetra-acetic acid TBE buffer using a CHEF Mapper apparatus (Bio-Rad, Hercules, CA, USA) at a potential of 6 V/cm pulsed from 5 to 35 s for 22 h at 14 °C [18]. The gels were stained with ethidium bromide and photographed under ultraviolet light. The *Spe*I restriction profiles were initially compared by visual inspection and isolates were considered to be closely related if they showed differences of less than three bands [19]. Computer-assisted analysis using BioNumerics software (Applied Maths, Sint-Martens-Latem, Belgium) was also performed. Cluster analysis was performed by the unweighted pair group method with mathematical averaging, and DNA relatedness was calculated using the band-based Dice coefficient with a tolerance setting of 1.0 % and 1.0 % optimization setting for the whole profile [20]. Isolates were considered to belong to the same cluster if the similarity coefficient was >80 % [21].

## Results

### Patient characteristics

In total, isolates were collected from 71 patients admitted to MacKay Memorial Hospital with multidrug-resistant *P. aeruginosa* infections. The male-to-female ratio was 44:27 (males, 61.97 %; 44/71). The age distribution of the male population was 73.80 ± 12.64 years; the age distribution of the female population was 75.00 ± 15.69 years. The overall mortality rate was 32.39 % (23/71), 31.82 % (14/44) in males and 33.33 % (9/27) in females.

The sources of multidrug-resistant *P. aeruginosa* infections were bacteremia (21/71; 29.58 %), urinary tract infection (18/71; 25.35 %), respiratory infection (13/71; 18.31 %), wound infection (8/71; 11.27 %), tip of catheters (5/71; 7.04 %), drain discharge (4/71; 5.63 %), ascites (1/71; 1.41 %), and pleural effusion (1/71; 1.41 %).

### Estimated glomerular filtration rate (eGFR) and creatinine (Cr) level

57.75 % (41/71) of the patients in this study had renal insufficiency. In total, 42.25 % (30/71) of patients were classified as eGFR level 1 (eGFR ≥60 mL/min), 9.86 % (7/71) were placed in eGFR group 2 (30 mL/min ≤ eGFR < 60 mL/min), and 47.89 % (34/71) belonged to eGFR group 3 (eGFR <30 mL/min). When classified by the creatinine (Cr) levels, 49.30 % (35/71), 15.49 % (11/71), and 35.21 % (25/71) of patients were in Cr level 1 (Cr <1.5 mg/dL), the Cr level 2 (1.5 mg/dL ≤ Cr < 3 mg/dL), and the Cr level 3 (Cr ≥3 mg/dL) groups, respectively.

### Resistance of the isolates to antibiotic monotherapy and combinations in vitro

Of the 71 multidrug-resistant *P. aeruginosa* isolates collected, 85.92 % (61/71) were susceptible to amikacin (MIC ≤16 mg/L); none (0/71) was susceptible to ceftazidime (MIC ≤8 mg/L), imipenem (MIC ≤2 mg/L) or meropenem (MIC ≤2 mg/L). Only 1.41 % (1/71) were susceptible to doripenem (MIC ≤2 mg/L), and 98.59 % (70/71) were susceptible to colistin (MIC ≤2 mg/L). There was no significant difference in the MICs of most monotherapies compared to the combined therapies, as shown in Table 1. These combination therapies included 0.03–128 mg/L ceftazidime plus 4 mg/L tazobactam, 8 mg/L phosphomycin, or 8 mg/L sulbactam respectively. The three carbapenems (imipenem, meropenem, and doripenem) with various concentrations from 0.03 to 128 mg/L were included in the combined therapies, as shown in Table 1.

**Table 1:** MIC reduced fold-change of the multidrug-resistant *P. aeruginosa* isolates to various antibiotic combinations

Antibiotic combinations	0.03–128 mg/L ceftazidime	0.03–128 mg/L imipenem	0.03–128 mg/L meropenem	0.03–128 mg/L doripenem
4 mg/L tazobactam	No difference^a^	No difference^a^	No difference^a^	No difference^a^
8 mg/L phosphomycin	No difference^a^	No difference^a^	No difference^a^	No difference^a^
8 mg/L sulbactam	No difference^a^	No difference^a^	No difference^a^	No difference^a^

No difference^a^: No significant difference in the MIC reduced fold-change

Rifampicin alone was not effective (MICs ranging from 16 to 128 mg/L) against any of the 71 isolates. However, 0.03–128 mg/L imipenem + 20 mg/L rifampicin, 0.03–128 mg/L meropenem + 20 mg/L rifampicin, and 0.03–128 mg/L doripenem + 20 mg/L rifampicin had lower MICs compared to each individual carbapenem alone against multidrug-resistant *P. aeruginosa* clinical isolates. Imipenem + 20 mg/L rifampicin showed good activity, similar to that of meropenem + 20 mg/L rifampicin and doripenem + 20 mg/L rifampicin. Each carbapenem combined with 20 mg/L rifampicin exerted synergy in vitro, indicating that carbapenems combined with 20 mg/L rifampicin may represent a potential combination therapy against highly multidrug-resistant *P. aeruginosa* infections.

### Carbapenemase-producing isolates

Six of the 71 isolates (8.45 %) were also positive for the Carba NP test. Subsequently, PCR and sequencing indicated that all isolates positive for the Carba NP test harboured the *bla*_VIM_ gene, with 7.04 % (5/71) having the *bla*_VIM-2_ gene and 1.41 % (1/71) with the *bla*_VIM-3_ gene.

### PFGE analysis of the P. aeruginosa isolates without Carbapenemase-producing gene

**Fig. 1:**
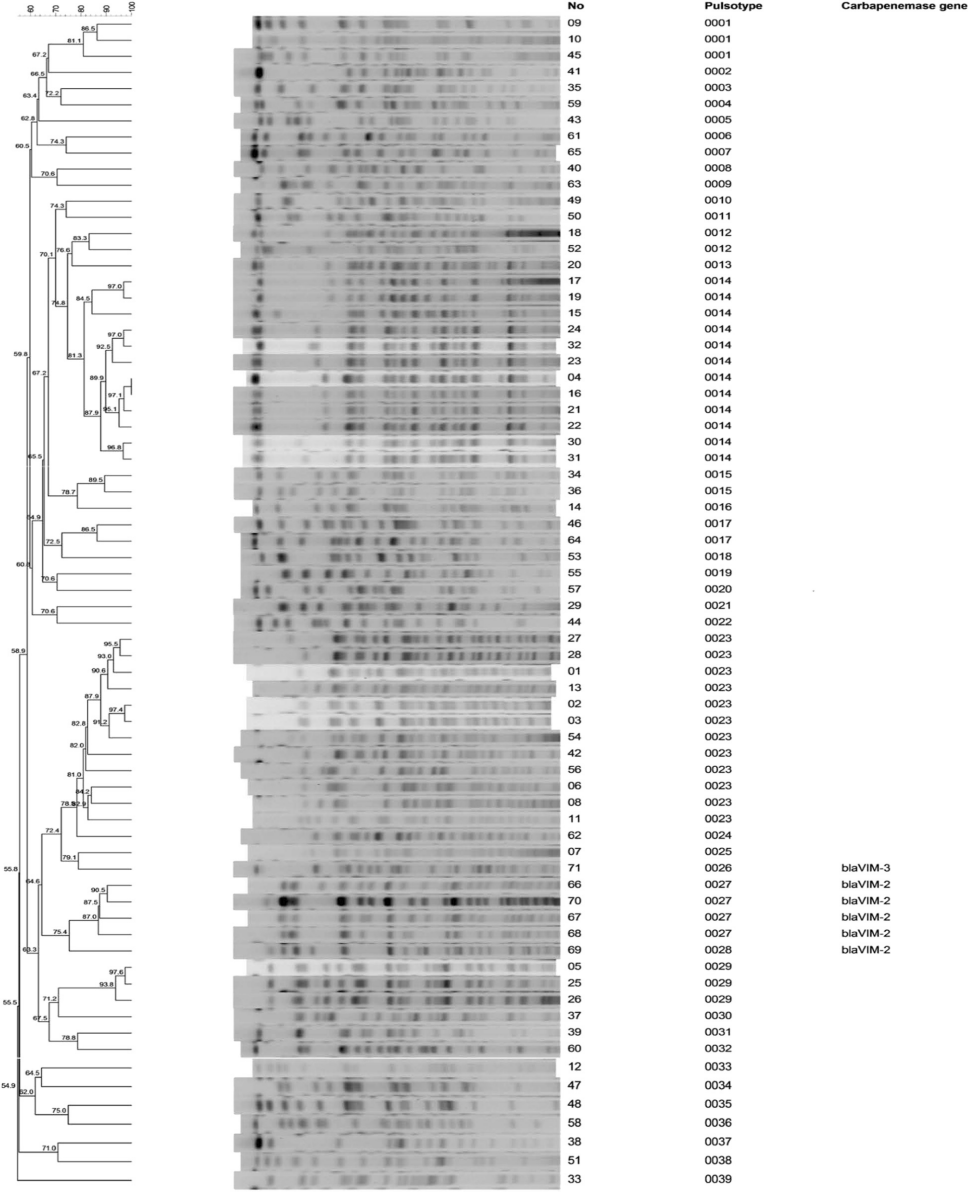
The PFGE analysis of 71 *Pseudomonas aeruginosa* isolates

The similarity of all 71 multidrug-resistant *P. aeruginosa* isolates with or without the *bla*_VIM_ gene was demonstrated in Fig. 1. Thirty-six PFGE patterns were classified from 65 multidrug-resistant *P. aeruginosa* isolates without the *bla*_VIM_ gene. The remaining six isolates with *bla*_VIM_ genes belonged to other three pulsotypes; the results were demonstrated in Fig. 1.

### Antibiotic combination for P. aeruginosa isolates with and without carbapenemase-producing gene

Excluding six *bla*_VIM_-producing isolates, there remained 65 isolates from 71 multidrug -resistant *P. aeruginosa*. The percentage of the 65 multidrug-resistant *P. aeruginosa* isolates for which combined treatment with 20 mg/L rifampicin resulted in lower MICs than imipenem, meropenem or doripenem alone is shown in Table 2. The isolates are classified by the presence or absence of the *bla*_VIM_ determinant and shown as the percentage of NR (non-resistant) or S (sensitive) isolates for each carbapenem in the presence or absence of rifampicin. No significant differences in the percentage of NR (non-resistant) or S (sensitive) isolates were observed between imipenem and rifampicin, meropenem and rifampicin, and doripenem and rifampicin compared to the individual carbapenems alone in the six isolates with the *bla*_VIM_ determinant.

**Table 2:** Percentage of the multidrug-resistant *P. aeruginosa* isolates that were resistant, non-resistant, sensitive to imipenem, meropenem and doripenem in the presence and absence of 20 mg/L rifampicin

	Monotherapy			Combinations with RIF		
	IMP	MEM	DOR	IMP	MEM	DOR
(a) *P. aeruginosa* isolates without the carbapenemase (*bla*_VIM_) gene (65 isolates)						
Resistant^a^	100 % (65/65)	95.38 % (62/65)	86.15 % (56/65)	13.85 % (9/65)	43.08 % (28/65)	15.38 % (10/65)
Non-resistant^b^	0 % (0/65)	4.62 % (3/65)	13.85 % (9/65)	86.15 % (56/65)	56.92 % (37/65)	84.62 % (55/65)
Sensitive^c^	0 % (0/65)	0 % (0/65)	1.54 % (1/65)	73.85 % (48/65)	47.69 % (31/65)	47.69 % (31/65)
(b) *P. aeruginosa* isolates with the carbapenemase (*bla*_VIM_) gene (6 isolates)						
Resistant^a^	100 % (6/6)	100 % (6/6)	100 % (6/6)	83.33 % (5/6)	83.33 % (5/6)	66.67 % (4/6)
Non-resistant^b^	0 % (0/6)	0 % (0/6)	0 % (0/6)	16.67 % (1/6)	16.67 % (1/6)	33.33 % (2/6)
Sensitive^c^	0 % (0/6)	0 % (0/6)	0 % (0/6)	0 % (0/6)	0 % (0/6)	0 % (0/6)

^a^Resistant (MIC >4 mg/L), ^b^Non-resistant (MIC ≤4 mg/L), ^c^Sensitive (MIC ≤2 mg/L)

*VIM* Verona integron-encoded metallo-β-lactamase, *IPM* imipenem, *MEM* meropenem, *DOR* doripenem, *RIF* rifampicin

However, imipenem + 20 mg/L rifampicin was the most effective combined therapy in vitro (versus any other carbapenem combination) against the 65 multidrug-resistant *P. aeruginosa* isolates that did not harbour the *bla*_VIM_ determinant. None of the 65 isolates without the *bla*_VIM_ determinant were sensitive to imipenem alone whereas 86.15 % (56/65) were non-resistant to imipenem combined with 20 mg/L rifampicin (Table 2).

**Fig. 2:**
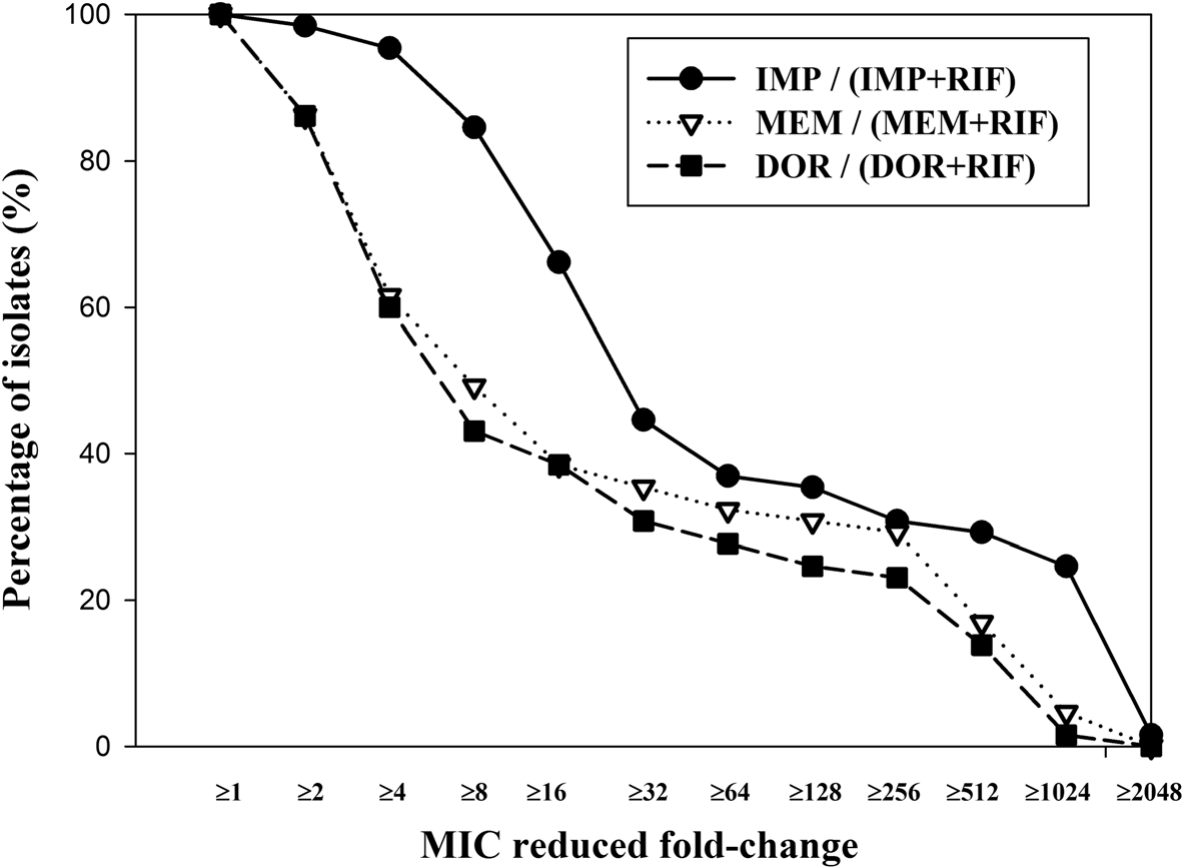
Percentage of the 65 multidrug-resistant *P. aeruginosa* isolates for which combined treatment with 20 mg/L rifampicin reduced the MIC compared to imipenem, meropenem or doripenem alone

Figure 2 shows the percentages of the 65 multidrug-resistant *P. aeruginosa* isolates without the *bla*_VIM_ determinant for which combined therapy with rifampicin resulted in lower MICs compared to imipenem, meropenem or doripenem alone. In accordance with a previous report [10] and as expected, imipenem + 20 mg/L rifampicin was confirmed as the most effective therapy against the multidrug-resistant clinical *P. aeruginosa* isolates in vitro.

### Phenotyping detection of hyperexpression of efflux pumps and cephalosporinase activity

MIC values of carbapenem agents were considerably reduced in the presence of the efflux inhibitor PaβN. Application of PaβN to multidrug-resistant *P. aeruginosa* isolates resulted in ≥2-fold decrease in MIC values for 95.38 % (62/65) of the isolates for imipenem, 87.69 % (57/65) of the isolates for meropenem, and 96.92 % (63/65) of the isolates for doripenem. This efflux pump inhibitor (PAβN) showed greater inhibitory activity when combined with imipenem, lowering 46.15 % (30/65), 35.38 % (23/65), 6.15 % (4/65), 6.15 % (4/65), 0 % (0/65) and 1.54 % (1/65) of the MIC values by 2-fold, 4-fold, 8-flod, 16-fold, 32-fold and 64-fold dilution, respectively. This efflux pump inhibitor (PAβN) showed greater inhibitory activity when combined with meropenem, lowering 12.31 % (8/65), 32.31 % (21/65), 41.54 % (27/65), 6.15 % (4/65), 6.15 % (4/65), 0 % (0/65) 0 % (0/65) and 1.54 % (1/65) of the MIC values by 1-fold, 2-fold, 4-fold, 8-flod, 16-fold, 32-fold, 64-fold and 128-fold dilution, respectively. This efflux pump inhibitor (PAβN) showed greater inhibitory activity when combined with doripenem, lowering 3.07 % (2/65), 27.69 % (18/65), 41.54 % (27/65), 20.00 % (13/65) and 7.69 % (5/65) of the MIC values by 1-fold, 2-fold, 4-fold, 8-flod and 16-fold dilution, respectively. It is noteworthy that a greater inhibitory effect was observed for imipenem, meropenem and doripenem when both efflux pump inhibitor (PAβN) and AmpC inhibitor (cloxacillin) were combined [100 % (65/65), 95.38 % (62/65) and 98.46 % (64/65) inhibition by ≥2-fold dilution].

### Antibiotic combination for isolates with oprD gene mutation

The 65 multidrug-resistant *P. aeruginosa* isolates without the *bla*_VIM_ determinant were screened for *oprD* gene mutations, and 21 isolates were classified as having a frameshift mutation while 39 isolates were classified as having premature stop codon mutation. Only five isolates were without an *oprD* mutation.

**Fig. 3:**
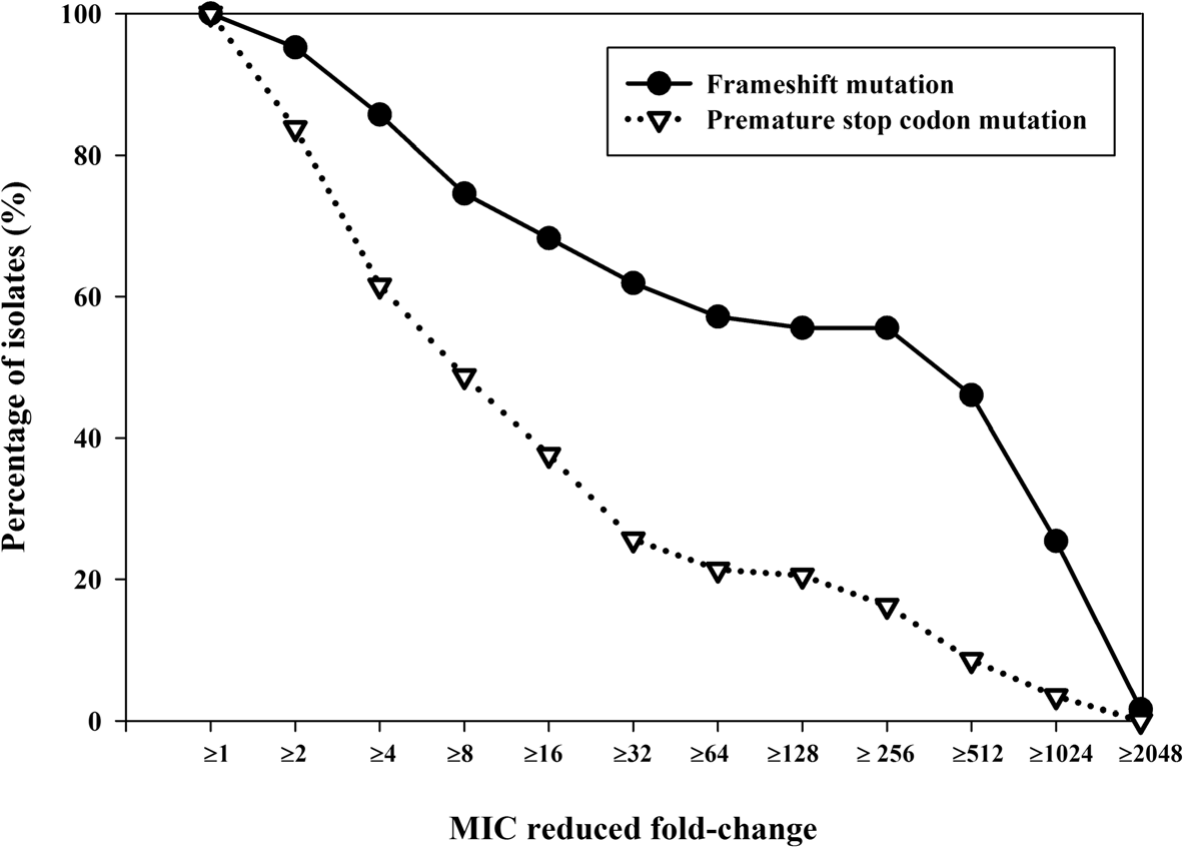
Percentage of the 60 multidrug-resistant *P. aeruginosa* isolates for which combined treatment with 20 mg/L rifampicin reduced the MIC compared to imipenem, meropenem or doripenem alone, stratified by the type of porin mutation (the 21 isolates with a frameshift porin mutation; the 39 isolates with a premature stop codon porin mutation)

The percentages of isolates with each type of *oprD* gene mutation for which combined therapy with 20 mg/L rifampicin resulted in a lower MIC than the carbapenem alone is shown in Fig. 3. Combined therapy with rifampicin resulted in lower MICs in isolates with the frameshift *oprD* mutation than with the premature stop codon *oprD* mutation.

**Fig. 4:**
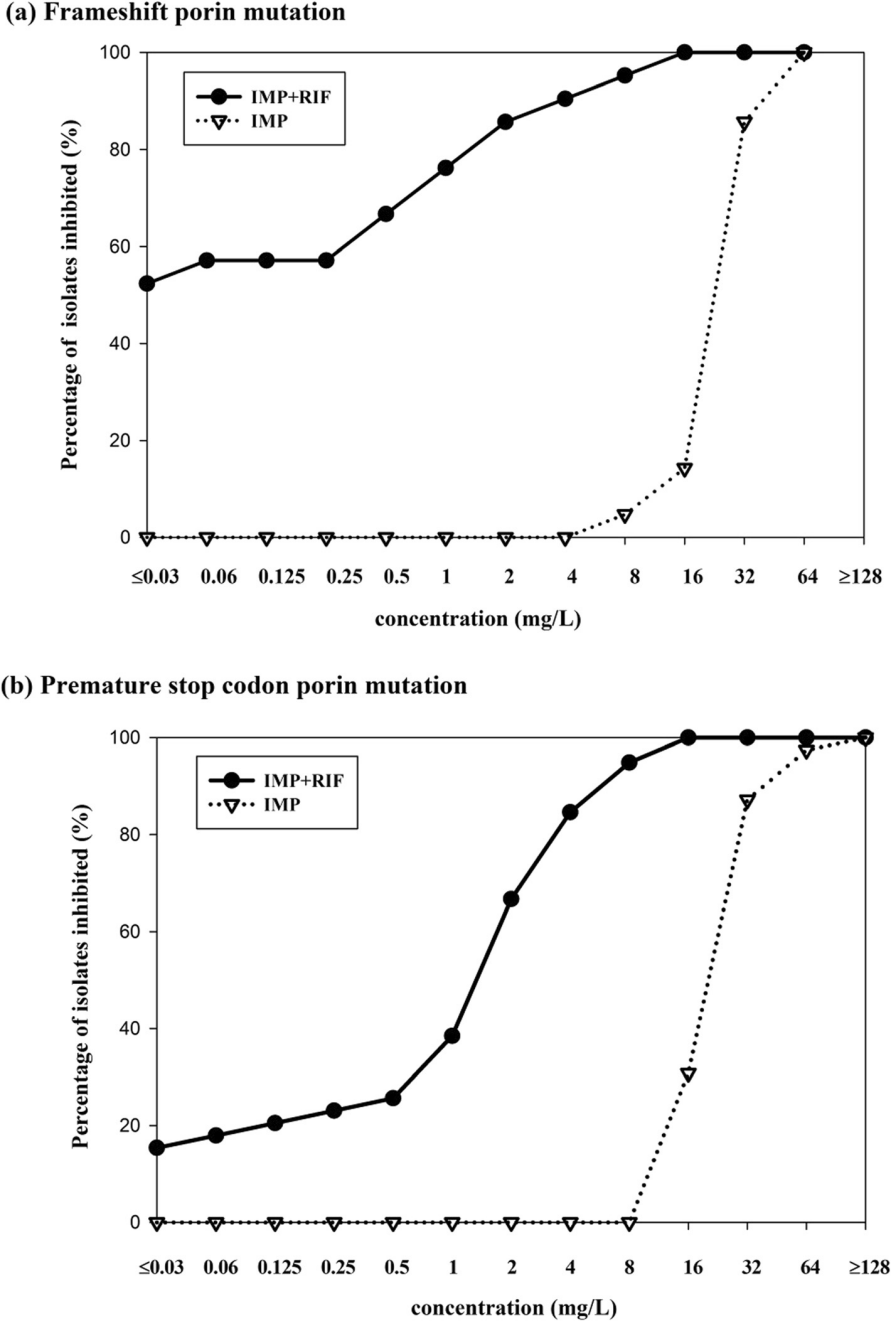
MIC values of multidrug-resistant *P. aeruginosa* isolates to imipenem between with or without 20 mg/L rifampicin, stratified as (**a**) The 21 isolated with a frameshift porin mutation; (**b**) The 39 isolates with a premature stop codon porin mutation

Figure 4a compares the MIC values for imipenem with and without 20 mg/L rifampicin in the 21 multidrug-resistant *P. aeruginosa* isolates with an *oprD* frameshift mutation. Figure 4b presents the MIC values for imipenem with and without rifampicin in the 39 multidrug-resistant *P. aeruginosa* isolates with an *oprD* premature stop codon mutation. Overall, the combined therapy had the greatest synergistic effect in the multidrug-resistant *P. aeruginosa* isolates with the *oprD* frameshift mutation and lower synergistic effect in the isolates with the *oprD* premature stop codon mutation.

## Discussion

Colistin is commercially available for clinical use; however, shortly after it was introduced clinically, reports of nephrotoxicity led to a significant decline in its use [5]. Therefore, an alternative treatment for multidrug-resistant *P. aeruginosa* infections is required to avoid the acute kidney injury associated with colistin treatment, especially in patients with renal insufficiency. The treatment options for multidrug-resistant *P. aeruginosa* infections are limited and combination therapy with other antimicrobial agents has often been suggested as a potential strategy. In particular, synergism between colistin + rifampicin has been demonstrated in several studies and the addition of a carbapenem to this regimen may be an option, despite the apparent resistance of multidrug-resistant *P. aeruginosa* [22]. However, as yet there is no evidence-based support for most combination therapies against carbapenem-resistant Gram-negative bacteria including colistin/carbapenem combination therapy [23].

The aims of this study were to search for the most effective colistin-free combinations of antibiotics against multidrug*-*resistant *P. aeruginosa* isolates in vitro and investigate the effect of specific mutations in the isolates without carbapenemase-producing genes (i.e., the porin frameshift mutation and premature stop codon mutation) on combined therapy in multidrug-resistant *P. aeruginosa* clinical isolates.

Carbapenems have different levels of activity against *P. aeruginosa* isolates. In vitro studies by Kanj et al. [12] showed that doripenem had the lowest MICs, followed by meropenem and imipenem. Goyal et al. reported that doripenem had an 84.2-fold lower MIC towards *P. aeruginosa* isolates (0.38 mg/L) than meropenem (>32 mg/L) [24]. In agreement with these previous results, doripenem had lower MICs than meropenem in the 71 multidrug-resistant *P. aeruginosa* isolates. However, 65 of the 71 multidrug-resistant *P. aeruginosa* isolates had doripenem MIC values >2 mg/L, with a high percentage of isolates non-susceptible to imipenem, meropenem and doripenem.

It is widely accepted that rifampicin should not be used as a monotherapy in order to avoid the development of rifampicin resistance [25]. In addition, Morris et al. reported that the MICs for rifampicin in most aerobic gram-negative bacilli were <12 mg/L, although MICs as high as 32 mg/L have been observed for *P. aeruginosa* [26]. Several lines of evidence in this study support these previous reports. The MICs for rifampicin were high in the *P. aeruginosa* isolates: the frequency distribution of the MICs for the 65 multidrug-resistant *P. aeruginosa* isolates was as follows: 13.85 %, 16 mg/L; 70.77 %, 32 mg/L; 13.85 %, 64 mg/L; and 1.54 %, 128 mg/L. Therefore, we investigated whether combined treatments could effectively inhibit multidrug-resistant *P. aeruginosa*.

Rifampicin can inhibit DNA-dependent RNA polymerase activity in susceptible *Mycobacterium tuberculosis* organisms [26]. Majewski et al. previously demonstrated that in vitro synergism or an additive interaction between rifampicin and imipenem occurred in *A. baumannii* strains showing resistance to imipenem [25]. In agreement with the data in this study, imipenem + 20 mg/L rifampicin, meropenem + 20 mg/L rifampicin, and doripenem + 20 mg/L rifampicin resulted in significantly lower MICs than the individual monotherapies alone. The performance of imipenem + 20 mg/L rifampicin combination was especially well.

An unexpected finding in this study was that the imipenem + rifampicin combination only showed bacteriostatic effects against *P. aeruginosa* isolates in vitro, and was not any more effective (than the individual monotherapies) against the six isolates harbouring the *bla*_VIM_ determinant. Therefore, we further investigated the activity of carbapenem + 20 mg/L rifampicin against the 65 *P. aeruginosa* isolates that did not harbour the *bla*_VIM_ gene.

A number of studies have found that the most prevalent intrinsic mechanism of multidrug-resistance in *P. aeruginosa* is inactivation of *oprD* [1, 3, 27]. Riera et al. revealed that imipenem resistance was driven by *oprD* inactivation, while *ampC* overexpression and, in particular, efflux pump hyperproduction had a lower impact on the activity of doripenem compared to meropenem among *P. aeruginosa* [27]. Vatcheva-Dobrevska et al. revealed that nearly all of 29 multidrug-resistant *P. aeruginosa* isolates (97 %) lacked OprD production, whereas only five isolates (17.24 %) overexpressed *ampC* [28]. Fournier et al. demonstrated that the porin OprD was lost in 94 (86.2 %) of isolates [3]. Castanheira et al. illustrated that *oprD* decrease/loss was the most prevalent intrinsic mechanism of carbapenem-resistance (94.9 % of *P. aeruginosa* isolates), followed by *ampC* overexpression (44.4 %) [1]. In line with these previous reports, 92.31 % (60/65) of the isolates tested in this study had *oprD* mutations.

To our knowledge, this is the first study designed to compare the combined activities of imipenem + rifampicin in multidrug-resistant *P. aeruginosa* concerning the types of porin mutations. We evaluated the efficacy of imipenem + rifampicin in isolates with porin frameshift mutation and premature stop codon mutation. Imipenem combined with 20 mg/L rifampicin was significantly more effective in the isolates with the porin frameshift mutation.

Our results and those of others clearly demonstrate the in vitro efficacy of the imipenem + rifampicin combination [10], which may be due to a synergistic effect against multidrug-resistant *P. aeruginosa* isolates with porin mutations and without *bla*_VIM_ producing genes. However, we cannot explain why the combination of imipenem + rifampicin exhibited a significantly higher efficacy in the isolates with a porin frameshift mutation. Interestingly, we also observed that the addition of 10 mg/L rifampicin to different concentrations of imipenem, meropenem or doripenem did not reduce MIC in the 71 multidrug-resistant *P. aeruginosa* clinical isolates.

This study provides valuable in vitro data on the MICs of various combinations of antibiotics on multidrug-resistant clinical *P. aeruginosa* isolates. However, the clinical significance of these findings needs to be evaluated. Our data indicates that imipenem + 20 mg/L rifampicin represents a promising alternative combination therapy for patients with multidrug-resistant *P. aeruginosa* infections; the use of such therapy obviates the need for colistin and the potential nephrotoxicity associated with its use, showing promise for patients with existing renal insufficiency. The combination of imipenem and rifampicin warrants further laboratory and clinical trials.

For providing quick clinical identification, we suggest that the Carba NP test should be used initially to screen for isolates harbouring carbapenemase-producing genes, and that rifampicin + imipenem combination therapy be used only for infections caused by multidrug-resistant *P. aeruginosa* strains without the *bla*_VIM_ determinant. The combination of rifampicin + imipenem demonstrated good efficiency in vitro against multidrug-resistant *P. aeruginosa* isolates that do not harbour the *bla*_VIM_ resistance gene, especially in isolates with a frameshift porin mutation. We must highlight the inherent limitations of this study in terms of its observational design and limited sample size.

## Conclusions

The combination of rifampicin + imipenem demonstrated good efficiency in vitro against multidrug-resistant *P. aeruginosa* isolates that do not harbour the *bla*_VIM_ resistance gene, especially in isolates with a frameshift porin mutation. Carba NP test is a very useful tool to screen for *P. aeruginosa* isolates that may be susceptible to the rifampicin + imipenem combination therapy, and can be easily and rapidly performed in most medical facilities. Imipenem + rifampicin could be an alternative treatment for multidrug-resistant *P. aeruginosa* infections. Such combination therapy avoids the risk for acute kidney injury-induced by colistin, which is especially important in patients with renal insufficiency.

## Abbreviations

CLSI: Clinical and Laboratory Standards Institute
Cr: Creatinine
eGFR: Estimated glomerular filtration rate
MDR: Multidrug resistant
MIC: Minimal inhibitory concentration
PCR: Polymerase chain reaction
PFGE: Pulsed-field gel electrophoresis
